# Combining electrochemical and quantitative elemental analysis to investigate the sulfur poisoning process of ceria thin film fuel electrodes†

**DOI:** 10.1039/d1ta06873c

**Published:** 2021-12-22

**Authors:** C. Herzig, J. Frank, A. Nenning, M. Gerstl, A. Bumberger, J. Fleig, A. K. Opitz, A. Limbeck

**Affiliations:** TU Wien, Institute of Chemical Technologies and Analytics Getreidemarkt 9/164 I^2^AC 1060 Vienna Austria alexander.opitz@tuwien.ac.at andreas.limbeck@tuwien.ac.at; TU Wien, Joint Workshop, Technical Chemistry Vienna Austria

## Abstract

This work deals with the effect of sulfur incorporation into model-type GDC thin films on their in-plane ionic conductivity. By means of impedance measurements, a strongly deteriorating effect on the grain boundary conductivity was confirmed, which additionally depends on the applied electrochemical polarisation. To quantify the total amount of sulfur incorporated into GDC thin films, online-laser ablation of solids in liquid (online-LASIL) was used as a novel solid sampling strategy. Online-LASIL combines several advantages of conventional sample introduction systems and enables the detection of S as a minor component in a very limited sample system (in the present case 35 μg total sample mass). To reach the requested sensitivity for S detection using an inductively coupled plasma-mass spectrometer (ICP-MS), the reaction cell of the quadrupole instrument was used and the parameters for the mass shift reaction with O_2_ were optimised. The combination of electrical and quantitative analytical results allows the identification of a potential sulfur incorporation pathway, which very likely proceeds along GDC grain boundaries with oxysulfide formation as the main driver of ion transport degradation. Depending on the applied cathodic bias, the measured amount of sulfur would be equivalent to 1–4 lattice constants of GDC transformed into an oxysulfide phase at the material's grain boundaries.

## Abstract 2

This work deals with the effect of sulfur incorporation into model-type GDC thin films on their in-plane ionic conductivity.

## Graphical abstract

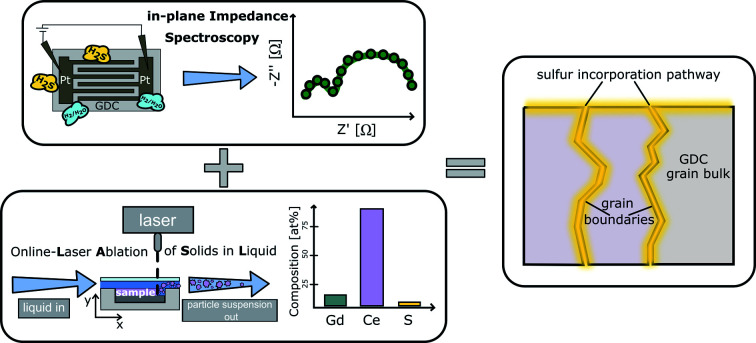

## 1. Introduction

Determination of S in bulk materials as well as in thin films or nanomaterials is of increasing interest due to the wide variety of physical, optical, electrochemical, or mechanical properties of metal sulfides, oxysulfides, or oxides interacting with H_2_S containing atmospheres.^2–4^ Usually, for all these functional materials, there is a strong link between elemental composition and the desired properties. Therefore, a precise determination of the composition during the preparation and optimization process is advantageous. However, the requirements for quantitative sulfur analysis are very different.

Gadolinium doped ceria (GDC) is a promising novel anode material for solid oxide fuel cells (SOFCs), with increased H_2_S poisoning resilience being one of its advantages. This benefit can be improved by using dopants^5,6^ or tailored fabrication procedures to influence the architecture of GDC thin films.^7^ Under certain conditions, however, also the performance of GDC anodes degrades during operation with fuels containing H_2_S in the ppm range.^8–10^ In recent studies, the incorporation of sulfur into GDC was discussed as a potential degradation mechanism, but many details such as the total incorporated S amount and the pathway of sulfur uptake remained open questions. In this study, the focus is laid onto the quantification of S in gadolinium doped ceria (GDC) thin films, because this material is used as a high performing alternative anode material in SOFCs, which are very promising electrochemical devices for a sustainable energy supply in the 21^st^ century.^11–13^ Gaseous S species are possible contaminations in the fuel gas, causing undesired losses of the electrochemical performance thus hampering the long-term stability of SOFCs. Though GDC-based anodes are known to have some sulfur poisoning resilience especially under anodic polarization (*i.e.* SOFC operation under load), a sulfur uptake by GDC and a related long-term poisoning effect was reported for more reducing conditions.^8,14^ Thus, there is a big interest in new analytical techniques for S determination in GDC to understand this degradation process.

A typical approach to investigate the elemental composition of solid samples is the use of X-ray-based analysis techniques, such as X-ray fluorescence (XRF), or energy dispersive X-ray spectroscopy (EDX), because both methods are well-established analytical techniques and work with standard-less quantification models. If the investigated sample is not a bulk material, but a thin film with about 100 nm thickness, the sensitivity of both methods is limited, and possible interferences with signal from the substrate material might impede the quantification approach. X-ray photoemission spectroscopy (XPS) as an alternative technique can help to identify the nature of chemical bonds, but only in the very near surface region of the material and above the detection limit of about 1 at% or with increased relative standard deviation of more than 20%.^15,16^

Besides these X-ray based techniques, laser-ablation in combination with inductively coupled plasma-mass spectrometry (ICP-MS) is a common lab-based technique for the elemental analysis of solid materials.^17–19^ Especially the high sensitivity of mass spectrometers allows the determination of inorganic traces and ultra-traces, which is of course an advantage, if the sample amount is rather small and limited as it is the case for thin films. However, due to matrix effects in the laser ablation, particle transportation and ionisation steps, matrix-matched standards are usually required for a correct signal quantification,^17,18^ hampering the application of this technique for analysis of new materials. Recently, a novel solid sampling strategy called online-laser ablation of solids in liquid (online-LASIL) has been proposed, which provides precise information about the stoichiometry of thin films without the need of solid reference materials.^19,20^

In the present work, the effect of sulfur uptake from H_2_S containing atmospheres into model-type GDC thin films on their ionic conductivity was studied. We measured the in-plane impedance of GDC thin films after exposure to reducing atmospheres with varying H_2_S content, and separated the obtained resistive response into a grain bulk and grain boundary contribution. This way, we could prove that the deterioration of the ionic conductivity was a grain boundary effect. Quantification of the incorporated sulfur amount was performed by a novel online-LASIL-ICP-MS approach. In order to adapt this analysis method to the rather demanding conditions of the sample system, a number of optimizations were made. These measures – among others – include an adaption of the online-LASIL ablation cell design, an adjustment of the carrier solution composition, and the use of the instruments reaction cell to avoid polyatomic isobaric interferences. With these adaptations, the limit of quantification for sulfur in GDC thin films could be significantly reduced, thus being able to quantify the small amounts of sulfur present with sufficient accuracy. Together with the electrical data, the knowledge of the total sulfur amount allows a critical discussion of different sulfur incorporation scenarios. The measurement results strongly indicate the formation of an oxysulfide phase at the grain boundaries, while an accumulation of sulfur solely in the grain boundary core or an even distribution throughout the entire GDC film are very unlikely.

## 2. Experimental

### 2.1. Preparation of samples for measurement of ionic conductivity

For measurement of the in-plane ionic conductivity of Gd_0.10_Ce_0.90_O_1.95_ (GDC) thin films, samples were prepared similar to the procedure described in earlier studies.^21,22^ As electrically insulating substrates [0001]-oriented, polished sapphire single crystals (10 mm × 10 mm × 0.5 mm; Crystec; Germany) were used. On these substrates, GDC films were grown by pulsed laser deposition (PLD) at 600 °C substrate temperature (measured by a pyrometer). The PLD target was prepared by isostatic pressing (*ca.* 2 kbar) and sintering of commercially available GDC powder (Treibacher Industrie AG, Althofen, Austria). It was ablated with a 248 nm KrF excimer laser (Coherent COMPex Pro 201F) with 90 mJ pulse energy (measured on the target; resulting in 1.1 J cm^−2^), 5 Hz repetition rate in 0.04 mbar O_2_ atmosphere for a duration of 30 minutes yielding 200 nm film thickness on the substrate. After deposition, the GDC thin films were annealed at 1100 °C in air. Subsequently, interdigitating current collectors were prepared on top of the GDC films by sputter depositing Ti/Pt thin films (5/100 nm) and micro-patterning them by means of lift-off photolithography.

### 2.2. Impedance measurements of ionic conductivity

Characterisation of the in-plane ionic conductivity of GDC thin films was done in accordance with previously published work by 2-wire impedance measurements at temperatures between *ca.* 175 and 425 °C in air. These oxidising conditions were needed to avoid effects of an increased electron concentration in GDC, which can potentially lead to a changed ionic grain boundary conductivity.^21^

Before these in-plane impedance measurements, the GDC thin films were annealed for 1 h at 760 °C in atmospheres that are typical for SOFC anodes. Initially, this atmosphere contained 25 mbar H_2_ and 1 mbar H_2_O (balance gas was argon). Subsequently, the samples were quenched, the reducing atmosphere replaced by air, and the in-plane measurement of the GDC film's ionic conductivity was done under oxidising conditions as mentioned above. Subsequently, sulfur poisoning was done by annealing for 1 h at 760 °C in 25 mbar H_2_, 1 mbar H_2_O, and 40 ppm of H_2_S (balance Ar), followed by the in-plane measurement in air. This procedure was repeated once more with the reducing atmosphere containing 200 ppm H_2_S.

From the measured impedance spectra, the GDC bulk as well as grain boundary resistances were extracted by a complex non-linear least squares fit employing the equivalent circuit, which was suggested and justified in ref. 21–23. From these resistances the ionic grain bulk and grain boundary conductivities were obtained considering the film thickness, current collector geometry, and average grain size (which was about 25 nm, in accordance with previous work^21^).

For comparison of conductivity data, an impedance measurement in air was also conducted on a dense, macroscopic GDC pellet prepared from the same powder as the PLD target, which was equipped with symmetric platinum electrodes. Grain bulk and grain boundary resistances were separated using the brick layer model.^24^

### 2.3. Samples for ICP-MS quantification of polarization dependent sulfur incorporation

**Fig. 1:**
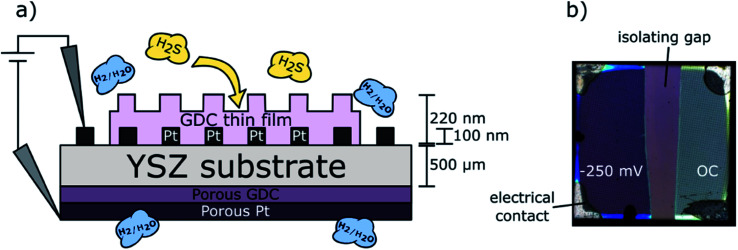
(a) Schematic cross section of GDC model-type sample; (b) top view micrograph of a degraded sample.

The model-type samples for quantification of sulfur in GDC consisted of thin films grown by PLD on 5 mm × 5 mm × 0.5 mm large [100]-oriented yttria stabilized zirconia (YSZ) single crystals (9.5 mol% Y_2_O_3_, CrysTec, Germany) by the procedure described above. The YSZ substrates act as oxide ion conducting electrolyte allowing operation as a full electrochemical cell in H_2_/H_2_O atmosphere with water splitting occurring at the GDC thin film electrode and hydrogen oxidation at the counter electrode. Hence, electrochemical polarisation of the GDC thin film became possible. To provide a homogeneous polarisation in electrochemical experiments, a Pt grid current collector was prepared on the YSZ electrolytes prior to the PLD process. This current collector was a 100 nm thick Pt film (with 5 nm Cr layer beneath for adhesion enhancement) magnetron sputtered onto the YSZ single crystals, which was subsequently micro-structured by photolithography and Ar-ion beam etching yielding a fine grid consisting of 5 μm thick Pt stripes with 15 μm distance between the stripes. This grid was split into two *ca.* 2 mm × 5 mm large stripes in order to obtain two electronically separated working electrodes. After structuring of the current collecting grid, the GDC layer was deposited. Thereby, the Pt grid it is embedded into the oxide electrode, thus obtaining model composite electrodes (almost) without accessible Pt/GDC triple phase boundary. Only the corners of the sample were shaded during the PLD process, to keep a small part of the Pt current collector free for contacting. The thickness of the prepared GDC thin films was also determined at these shadowed corners with a profilometer (DektakXT, Bruker, Massachusetts, USA). A schematic cross section of a sample is depicted in Fig. 1a.

It is at this point worth mentioning that we are well aware that the GDC film in this case grows slightly different as in case of sapphire substrates (used for in plane conductivity measurements). From previous work^8^ we already know that GDC deposited on top of the Pt current collector grows polycrystalline while the film part directly above the YSZ substrate is obtained strongly oriented. Nevertheless, the sample type with GDC model composite films grown on YSZ are sufficiently comparable to the GDC films deposited on sapphire to use them for quantifying the incorporated sulfur amount: (i) the film part grown directly on YSZ also contains 2D defects such as grain or domain boundaries, albeit with somewhat lower density as in case of sapphire substrate. (ii) Large parts of the polycrystalline GDC film grown above the Pt current collector can be electrochemically polarized, since GDC is a sufficiently fast ionic conductor – for more details see ref. 25.

Before working electrode preparation, a kinetically fast^26^ porous GDC counter electrode was deposited on the bottom side of the YSZ electrolyte. For this means, a suspension of GDC particles (250 nm average particle size, Treibacher Industrie AG) in commercial ink vehicle (Fuelcellmaterials, OH, USA) was spin-coated on the single crystal. Subsequently, the layer was dried at 120 °C and a Pt–GDC (50/50 vol%) composite layer followed by pure Pt on top were applied by brushing of corresponding particle suspensions. The counter electrode assembly was then sintered in air at 1150 °C for 3 h. The resulting layer thickness was 3 μm for the GDC layer and 5 μm for the porous Pt current collector.

For electrochemically assisted sulfur poisoning of the GDC thin films, the samples were placed into a quartz tube inside a tubular furnace, heated to 760 °C, and flushed with 25 mbar H_2_ + 1.25 mbar H_2_O in Ar. One half of the platinum current collector was contacted with Pt wires and negative bias voltages between −60 and −250 mV were applied. This cathodic polarization leads to a stronger reduction of the GDC films – at OCV, the gas phase *p*(O_2_) is 10^−22^ bar, and is further reduced to ∼10^−27^ bar at −250 mV. Results published in literature show that under more reducing conditions the amount of sulfur incorporated in ceria and GDC ceramics is significantly increased.^8,14^ The second half of the grid stayed at open circuit (OC) potential at the same time as it is shown in Fig. 1b. This way, the samples were annealed for 24 hours, after which 200 ppm H_2_S were introduced for another 24 hours while maintaining the polarization on one half of the GDC film. Finally, the sample was quenched with a cooling rate of 500 °C min^−1^.

### 2.4. ICP instrumentation

For elemental analysis of ablated thin films, a ThermoFisher mass spectrometer (iCAP QC, ThermoFisher Scientific, Bremen, Germany) equipped with a reaction cell (QCell flatapole) and a quadrupole mass filter was applied. Liquid sample introduction was achieved with an external peristaltic pump (Perimax 12, SPETEC, Erding, Germany), a PFA μ-flow nebulizer (Elemental Scientific, Omaha, USA) and a Peltier-cooled cyclonic spray chamber (Glass Expansion, Melbourne, Australia). The instrument was tuned on a daily basis to ensure maximum signal intensity for ^115^In, reduced oxide ion ratio (^140^Ce^16^O/^140^Ce < 0.02) and the amount of doubly charged ions (^138^Ba^2+^/^138^Ba) below 3%. The laser ablation process was carried out with a Tandem LIBS instrument (J200 Tandem LIBS, Applied Spectra, Freemont, CA) equipped with a frequency quadrupled Nd:YAG laser (266 nm, 4 nm pulse duration). Further details on instrument operation parameters can be found in Table 1. To validate the results obtained by online-LASIL-ICP-MS at least for the main components of the thin films, a scanning electron microscope (SEM, Quanta 200, FEI, USA) equipped with an energy dispersive X-ray spectrometer (EDX – Octane Pro Silicon Drift Detector, Ametek) was used.

**Table 1:** Instrument operation parameters for online-LASIL measurements

**Laser ablation system**	
Wavelength	266 nm
Pulse duration	4 ns
Repetition rate	20 Hz
Laser fluence	2.9 J cm^−2^
Spot size	160 μm
Scan speed	0.8 mm s^−1^

**Liquid carrier solution**	
Carrier 1	40 μmol L^−1^ sodium phosphate buffer solution, pH = 8
Carrier 2	20 vol% HNO_3_ + 1 ng g^−1^ In
Carrier solution flow rates	Carrier 1: 0.29 mL min^−1^
	Carrier 2: 0.46 mL min^−1^

**Plasma operation parameters**	
Cool gas flow	13.8 L min^−1^
Auxiliary gas flow	0.79 L min^−1^
Nebuliser gas flow	1.01 L min^−1^
RF power	1400 W

**Reaction cell**	
Reaction gas	10 vol% O_2_ in He
Reaction gas flow rate	1.3 mL min^−1^
Pole bias	13.81 V
CCT bias	−2 V

**Mass spectrometer settings**	
Dwell time	10 ms (In), 50 ms (Gd, Ce), 200 ms (SO)
Measured isotopes	^32^S^16^O, ^115^In, ^142^Ce, ^157^Gd

### 2.5. Online-LASIL ablation cell

**Fig. 2:**
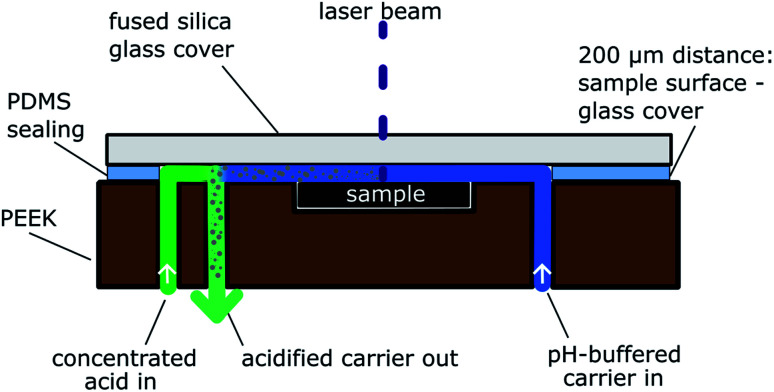
Schematic cross section of online-LASIL ablation cell.

The ablation cell used in this work is based on the design reported in detail in ref. 20, but was slightly adapted for this type of samples. The main body still consisted of a chemically resistant polymer (polyether-ether-ketone, PEEK) and possessed a cavity (0.5 mm) to house the sample, but three drillings for in- and outlets of the carrier solution were used. A schematic drawing of the cross section can be found in Fig. 2. One of the drillings is on one side of the sample and is used as the inlet for the carrier solution, which is in direct contact with the sample surface. The second drilling directly besides the sample acts as the outlet for the particle suspension. The third drilling further away from the sample is employed as a second inlet. This second inlet can be used to add acids and an internal standard to the particle suspension directly after the ablation process. With this setup it is ensured, that the sample surface is only in contact with a liquid, which does not cause any chemical reaction. This is an important advantage if the sample surface is pH-sensitive, but significant acidification of the particle suspension is necessary to avoid analyte dispersion or losses during transport to the detection unit. Sealing and fixation is achieved with a flexible polymer foil (polydimethyl-siloxane, PDMS), a fused silica window, and a metal ring with screws as reported in ref. 20.

### 2.6. Online-LASIL sampling and quantification procedure

For the online-LASIL process, the solid sample is placed in the cavity of the ablation cell and is sealed with the PDMS foil, the cover glass, and the fixation mechanism. Inlet one is started being flushed with 40 μmol L^−1^ buffer solution (carrier 1) prepared by mixing deionised water (18.2 MΩ cm^−1^) provided by Barnstead™ Easypure™ II and sodium phosphate buffer solution (20 mM, pH = 8, Fluka Analytical, Buchs, Switzerland). After the system is completely filled with the buffer solution, the second inlet is started to be flushed with 20 vol% nitric acid and 1 ng g^−1^ In (carrier 2) prepared using deionised water, concentrated nitric acid (65 mass%, EMSURE®), and In single element ICP-MS standard (1000 ng g^−1^, Certipure®, Merck, Germany). Indium was employed as an internal standard to correct for instrumental drifts and changes in the signal intensities. The flow rate of carrier 1 and 2 were determined to be 0.29 mL min^−1^ and 0.46 mL min^−1^ respectively. A PFA tube with 0.25 mm inner diameter (ID) serves as connection of the ablation cell with the liquid sample introduction system of the ICP-MS device.

For sample ablation, the cell itself was placed into the movable ablation chamber of the J200 LIBS instrument and a meander-like ablation pattern with the size of 2.1 × 0.5 mm^2^ was applied. The laser spot (160 μm) was entirely ablating this area within 15 s. The fluence (2.9 J cm^−2^) was adjusted to completely remove the GDC thin film in the first run. Complete ablation was verified by observing signal intensities of the substrate material (Y and Zr) and is important because the total GDC mass is necessary to determine the S content in the film afterwards.

Signal quantification was achieved with standard solutions varying in analyte concentration prepared by mixing 20 vol% nitric acid with certified single element standards of S, Ce (Specpure®, Alfa Aesar, ThermoFisher, Germany), and Gd (Certipure®, Merck, Germany). With the help of a six-way valve and a sample loop, an external calibration for absolute quantification could be determined. A detailed description of the calibration procedure and a flow-chart of the sample loop set-up can be found in the ESI.† The choice of isotopes used for quantification was partially based on preliminary experiments reported previously.^27^^32^S^16^O was observed to show the best signal to background ratio of all other plausible combinations of ^33^S, ^34^S, ^36^S and ^18^O, because ^32^S^16^O combines the most abundant isotopes of S and O. Possible isobaric interferences of ^156^Gd caused by ^140^Ce^16^O were prevented by using ^142^Ce, and ^157^Gd for quantification.

## 3. Results

### 3.1. In-plane ionic grain bulk and grain boundary conductivities and their response to H_2_S exposure

**Fig. 3:**
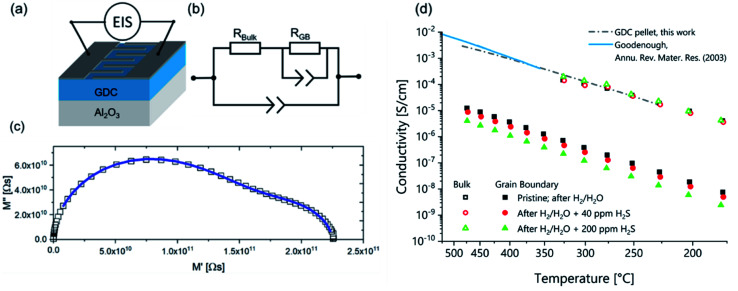
Ionic conductivity of GDC thin films: (a) sketch of the GDC thin film sample geometry with interdigitating electrodes. (b) Equivalent circuit used for CLNS fitting. (c) Impedance measurement (symbols) and CLNS fit (solid line) in the complex modulus plot. (d) Arrhenius diagram of the ionic grain and grain boundary conductivities. Open symbols show grain bulk and closed symbols grain boundary conductivities. The three different symbols/colours denote different degrees of H_2_S poisoning (ranging from no to 200 ppm H_2_S in the atmosphere; without applied bias voltage). The dash-dotted line shows GDC grain bulk conductivity measured on a macroscopic GDC pellet in this work, the solid line shows the total conductivity of macroscopic Gd_0.10_Ce_0.90_O_1.95_ taken from ref. 1.

With an interdigitating finger electrode design (sketched in Fig. 3a) and a modified brick-layer equivalent circuit model (shown in Fig. 3b), it is possible to measure the ionic grain bulk and grain boundary conductivities on GDC thin films, as already extensively elaborated in literature.^21–23^ A typical impedance spectrum recorded at 230 °C, together with the CLNS fit is plotted in Fig. 3c in a complex modulus plot. The modulus is calculated as *M* = *Z*/*ω* with *ω* being the angular AC frequency and *Z* the complex impedance. From this CLNS fit, we can calculate grain and grain boundary conductivities, which are shown in the Arrhenius plot in Fig. 3d. It can be clearly seen that the grain bulk experiences only minor if any conductivity changes upon exposure to H_2_S containing H_2_/H_2_O atmospheres (see open symbols). The grain boundary conductivity (closed symbols), however, significantly reacts on the H_2_S treatment, dropping by about a factor of five when comparing the virgin and the 200 ppm poisoned GDC thin films. This can be seen as a very strong indication that sulfide ions tend to preferentially penetrate the GDC grain boundaries while leaving the grain interior virtually unaffected.

For the sake of comparison, Fig. 3 also contains ionic conductivity data measured in air on macroscopic GDC polycrystals of identical doping. The data by Goodenough^1^ refers to the total conductivity, while in case of the GDC pellet characterized in the present work the grain bulk conductivity was separated from the grain boundary contribution. Since for 10 at% Gd doping the grain boundary resistance has only a small contribution to the total conductivity, both measurements yield very similar results. From the slope of the ionic grain bulk conductivity curve of the GDC pellet an activation energy of 0.66 ± 0.03 eV was extracted, which is in excellent agreement with literature.^28^ Since the ionic grain boundary conductivity of GDC thin films can significantly deviate from the value measured on macroscopic polycrystals for various reasons,^21^ a comparison of the grain boundary conductivities measured on GDC pellet and thin films was omitted here.

### 3.2. Electrochemically assisted sulfide incorporation into GDC

**Fig. 4:**
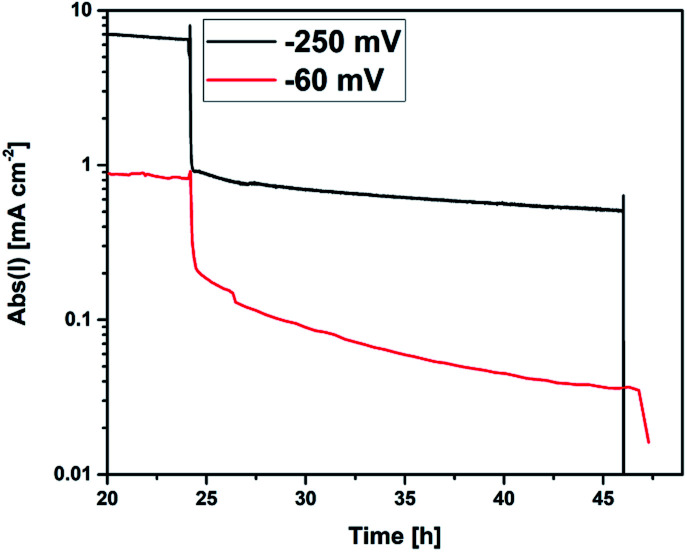
DC current recorded on the cathodically polarized half of the sample during the annealing process. Start of H_2_S exposure after 24 h.

As already mentioned above (Section 2.3), each sample was carrying two electrically separated macroscopic electrodes, one of which was cathodically polarized and the second remained at open circuit conditions during the H_2_S poisoning experiments. By recording the DC current of the polarised sample half, the performance degradation process could be monitored during the annealing process in H_2_S containing atmosphere. In Fig. 4, the measured currents for two differently polarized samples are depicted (water splitting at the working electrode and hydrogen oxidation at the counter electrode). After 24 h of pre-annealing, 200 ppm H_2_S were introduced into the atmosphere, leading to a significant and quick drop of the electrochemical current density. The current decreased to about 25% of the starting value within only a few minutes, which can be mainly explained by poisoning of catalytically active surface sites for the electrochemical reaction on the GDC surface.^8,29^ After this immediate drop, the degradation process continued on a much longer timescale, observable as a continuously decreasing current during the whole annealing time of 24 h. Incorporation of sulfide ions into GDC can be expected to mainly occur during this phase.^30^ After 24 h of H_2_S exposure, the experiment was stopped by quenching the sample while maintaining polarization. In Fig. 1b, a remarkable change in the colour of the thin film is observable on the left side of the sample after the degradation process, indicating that indeed both sides have experienced a clearly different treatment.

### 3.3. Optimization of ICP-MS measurement parameters

#### (a) Measurement of S using mass shift reaction

Polyatomic interferences at *m*/*z* = 32 impede a straightforward measurement of S on its most abundant isotope (^32^S: 95% relative abundance). The dominant polyatomic interference is ^16^O_2_^+^, but ^14^N^18^O^+^ and ^15^N^16^O^1^H^+^ could not be neglected in a liquid matrix containing significant amounts of nitric acid.^31^ If the instrument is equipped with a collision cell (Qcell), isobaric interferences may be removed by kinetic energy discrimination (KED mode) or by chemical reactions (CCT, reaction mode). In the first case, the Qcell is pressurized (few 10^−2^ mbar) with an inert gas or gas mixture (*e.g.*, 7 vol% H_2_ in He), which leads to energy losses caused by collisions of the analyte ions with the gas atoms/molecules. In the second case, the Qcell is operated with a reactive gas (*e.g.*, O_2_ or NH_3_) where collisions of the ions with the gas molecules possibly lead to reaction products with altered *m*/*z* ratios.^31^ In this work, 10 vol% O_2_ in He was used as reaction gas. If a possible reaction of the analyte ion with the reaction gas is exothermic, a corresponding polyatomic ion will be received. In the case of S^+^ the change in the reaction enthalpy Δ*H*_r_ is indeed negative (−26 kJ mol^−1^), but neither for O_2_^+^ nor for NO^+^ and NOH^+^.^31^ Thus, only S will experience a mass shift and can be detected as SO^+^ with *m*/*z* = 48, which is usually free of interferences if Ca and Ti are not present in the sample.

In a first optimization step, the appropriate collision cell mode was determined by comparing the limits of detection (LOQ) of both modes. In CCT mode, a LOQ of 10 ± 5 ng g^−1^ for ^32^S^16^O was obtained according to DIN 32645, which is 20 times lower than the value obtained in the KED mode. In the second optimization step, the flow rate of the reaction gas was adjusted to the liquid carrier flow rate and the expected S concentration in solution. Detailed information about the optimization process, the data facilitating, and the chosen parameters can be found in the ESI (Fig. S2†). This two-step process is necessary to reach utmost sensitivity.

#### (b) Reduction of memory effects

**Fig. 5:**
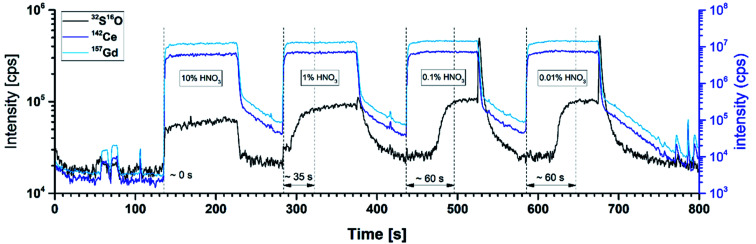
Transient signals for standards (0.01–10% HNO_3_) and washing solution (10% HNO_3_) showing a decreased memory effect for S in standards with high acid concentration.

Adsorption effects of analytes can bias the result of the measurement and lead to unpreferred long washout times necessary to reach signals at the background level. Thus, a preliminary set of experiments with different solutions was performed to test for the presence/absence of memory effects. Several additives such as ammonium phosphate ((NH_4_)_3_PO_4_), ammonium chloride (NH_4_Cl), ammonium acetate (NH_4_(CH_3_COO)), ammonium carbonate ((NH_4_)_2_CO_3_), boric acid, citric acid and varying nitric acid concentrations were investigated. All solid additives were used as solutions of 0.01 wt% in deionised water and were flushed through the whole online-LASIL system in alternation with liquid standard solutions. The transient signals were recorded and checked for delays in the signal increase of S compared to Ce, Gd or In. The most promising results can be seen in Fig. 5. Here, standard solutions containing 80 ng g^−1^ S, 4 μg g^−1^ Gd and 12.8 μg g^−1^ Ce with varying nitric acid concentrations were flushed through the whole online-LASIL setup including the ablation cell. After standard solution, a blank solution containing 10 vol% HNO_3_ was introduced to clean the system. The two blue lines representing the intensities for Ce and Gd reach very quickly a stable plateau with sharp edges, indicating that no retention of these elements occurred in the components of the LASIL system. The transient signal for S (black line), however, only follows this trend in a solution with 10 vol% nitric acid. As soon as the acid concentration is reduced, the start of the S plateau is delayed up to 60 s compared to the plateau of Ce and Gd. During this time, the analytes are adsorbed on the walls of the tubes or the surfaces of the ablation cell. With the introduction of the strong acidic washing solution the retained analytes are then released and a sharp intensity spike for S is observed at the end of the plateaus. These spikes are clearly visible in Fig. 5 at the end of the standard plateaus containing 0.1 and 0.01 vol% HNO_3_.

All other investigated additives are far less efficient in reducing this memory effect of S except of (NH_4_)_3_PO_4_. Unfortunately, this substance significantly increases the background on *m*/*z* = 48 because P is affected in the reaction cell as well and possibly forms ^15^P^16^O_2_^1^H^+^ or other polyatomic interferences.

As a consequence of this analyte washout experiments, an increased acid concentration of at least 5 vol% was chosen for all further experiments. With the optimized carrier solution composition ensuring the avoidance of S memory effects, calibration curves could be obtained for all elements in good quality even for S. Liquid standard solutions with varying analyte content ranging from 2 to 8 ng for S, 34 to 270 ng for Gd, and 100 to 740 ng for Ce were injected and flushed through the whole online-LASIL set-up including the ablation cell with a blank sample. To obtain a very good linearity also for the lowest S concentration level (2 ng), the signal to noise ratio (S/N) for SO was improved by increasing the dwell time for *m*/*z* = 48 to 200 ms as suggested in literature^32,33^ and intensively studied for the case of online-LASIL measurements herein.^27^ The transient signals were integrated and together with the known absolute analyte mass of the standards, regression functions were obtained. All elements showed good linearity with a correlation coefficient (COD) > 0.9986 and *F* values (ANOVA) of more than 1470.

#### (c) Adaptation of cell design for pH-sensitive samples

Pure, unpoisoned GDC thin films are in general chemically very inert and remain insoluble also in strongly acidic conditions. After the exposure to H_2_S in reducing atmosphere, however, the films became slightly soluble in acids, which prevented the use of an acidic carrier solution in direct contact with the sample surface. The acidity in the carrier solution would partially dissolve the thin film solely by flushing the carrier solution through the system. During the degradation process, S containing species of Ce and Gd were obviously formed exhibiting different chemical durability. Especially for Ce, various species containing O and S are known in literature (*e.g.*, Ce_2_O_2_S, Ce_2_S_3_)^34^ and an easy solubility for Ce_2_O_2_S in diluted acids is reported.^35^ Thus, the use of a buffered carrier solution was necessary to prevent dissolution of the soluble GDC-species produced in the degradation process. To pay attention to the results obtained in the preliminary memory-effect experiments without bringing the sample surface in direct contact with concentrated acidic liquid, the particle suspension was acidified directly after the ablation step to reduce the memory effect of S.

This delicate handling of pH sensitive samples was not possible with the 2-port ablation cell used in previous studies,^19,20,27^ but was only possible with a novel ablation cell design reported herein featuring two inlets for two different carrier solutions (see Fig. 2). The important aspect of this cell is the extremely short distance between the area of particle ablation and acidification (4 mm from centre of the sample to point of acidification). Due to the flow rate of the carrier solution, only a very limited time (<1 s for complete exchange of liquid in cell) for possible adsorption processes is available. In the case of simple design adaptions, for example when using a T-piece at the analyte outlet for mixing the buffered carrier solution with an acid, the distance between particle production and acid addition is much larger and memory effects cannot be avoided efficiently.

### 3.4. Quantitative analysis of H_2_S treated GDC thin films

To quantify the uptake of S into the GDC thin film, the optimized online-LASIL-ICP-MS approach was used to determine the elemental composition of the film. In total three model-type GDC thin film samples were prepared and degraded in H_2_S containing atmosphere consecutively. Please note that on each sample one half of the GDC thin film was polarized and the other one stayed at open circuit, since all samples were separated in two halves by a gap in the Pt grid for electrical contact (see Fig. 1b and Section 2.3). Three negative voltages were applied (−60, −100, −250 mV) throughout this study and three OC parts of the samples were produced. To improve statistics, at least three replicate measurements were conducted for each biased sample area. This was possible because only a small area of the sample (1.05 mm^2^) is ablated during one scan. Signal quantification was achieved with regression curves derived from liquid standard solutions. The obtained analyte masses in measures of ng for Gd and Ce were transformed into molar amounts by means of atomic weights to calculate the molar ratio of Gd and Ce (in measures of cation fraction, *cf.* %) in the thin film. In total 18 measurements of the three thin film samples revealed a cationic composition of the main components in the film of 87 ± 0.5 *cf.* % Ce and 13 ± 0.5 *cf.* % Gd. For additional verification, SEM-EDX measurements were conducted on the GDC films and could confirm the slight off-stoichiometry from the nominal cation fraction – see Table 2. Since, the sensitivity of the SEM-EDX instrument for S detection was not sufficient for all analysed GDC thin films, only the main components Ce and Gd were detectable.

**Table 2:** Resulting composition of all analysed GDC thin films obtained by online-LASIL-ICP-MS and SEM-EDX measurements

Analysis technique	Gd cation fraction [*cf.* %]	Ce cation fraction [*cf.* %]
Nominal value	10	90
Online-LASIL-ICP-MS	13 ± 0.5 (1 s, *n* = 18)	87 ± 0.5 (1 s, *n* = 18)
SEM-EDX	12 ± 0.8 (1 s, *n* = 6)	88 ± 0.8 (1 s, *n* = 6)

More important than the cationic composition is in this case the S content in the film. Because O is not accessible by ICP-MS, the total mass of the ablated thin film was calculated on the basis of the obtained masses of Gd and Ce and the nominal O stoichiometry (Ce_0.9_Gd_0.1_O_1.95_). The detailed formula for the calculation can be found in the ESI.†

**Fig. 6:**
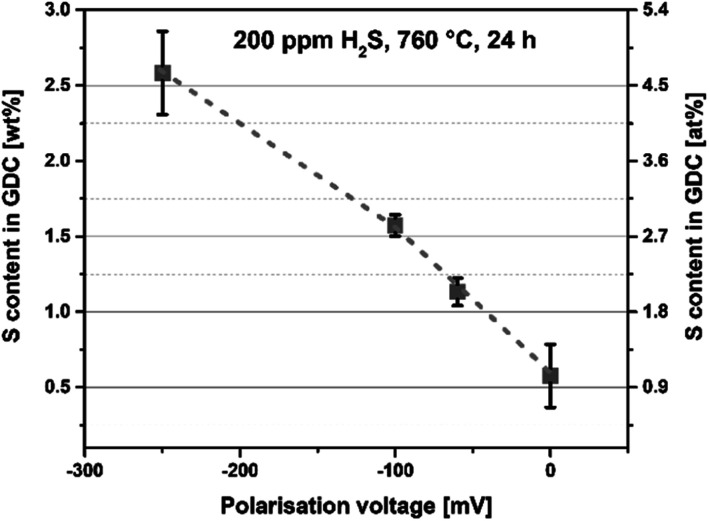
S content of degraded GDC thin films obtained by online-LASIL-ICP-MS given in measures of wt% or at% always based on either the total mass or the total moles of GDC obtained during the measurement.

With the calculated total film mass and the mass of S detected, the sulfur uptake was determined in measures of wt%. In Fig. 6 the amount of S uptake is plotted *versus* the applied bias voltage. Important to notice is an obvious correlation between the bias voltage and the S uptake. More negative bias voltage leads to more S incorporated into the thin film. Another interesting aspect in Fig. 6 is that also the unpolarised parts of all three samples show a certain S content.

Thus, the combination of electrochemical data and ICP results allows the following two important conclusions:

(i) In an atmosphere containing H_2_ : H_2_O in a ratio of about 25 : 1, a certain sulfide incorporation can also occur at 760 °C without additional bias, which is also in accordance with the observation of a changed ionic grain boundary conductivity upon H_2_S treatment (see Fig. 3). Moreover, this behaviour is thermodynamically favourable.^14,34^

(ii) Cathodic polarisation, which leads to an electrochemical reduction and thus a lower effective *p*(O_2_) within the GDC electrode,^36,37^ causes significantly enhanced sulfur uptake. This is also in line with the expected behaviour, since S-containing Ce–O phases are thermodynamically favourable under more reducing conditions and contain a high concentration of reduced Ce^3+^ ions.^14^

### 3.5. Additional analytical analysis

#### (a) Quantitative XRF measurements

X-ray fluorescence (XRF) is used as an alternative technique to verify the sulfur content in the GDC films obtained by online-LASIL-ICP-MS. For these measurements, the sample with one half being cathodically biased at −250 mV was used. In contrast to online-LASIL measurements, the probed area of the XRF measurements is rather large (circle, 10 mm in diameter) and a separation of the biased part of the sample from the unpolarized part is not possible. Instead, an averaged sulfur content for the whole sample of 2.9 at% in GDC is obtained. To be able to compare this value with the two results obtained by online-LASIL-ICP-MS for the biased and the OC part of the sample, an average over the entire sample area is calculated as a linear combination of both ICP results using the respective area fractions as weighting factors. The resulting area-weighted online-LASIL measured S content in GDC averaged over the whole sample is 2.6 ± 0.5 at% (*n* = 3). This is insignificantly different from the XRF measurement, hence confirming the reliability of the presented online-LASIL-ICP-MS approach (please note that the quantification of the XRF measurement relies on a mathematical model provided by the software of the semi-automatic XRF machine. For higher accuracy of the XRF measurement, a calibration using standards of known composition would be required, which is beyond the scope of the present study). Further details about the instrument used for the XRF measurement and the area-weighted S content calculation steps can be found in the ESI.†

#### (b) Qualitative TEM-EDX measurements

**Fig. 7:**
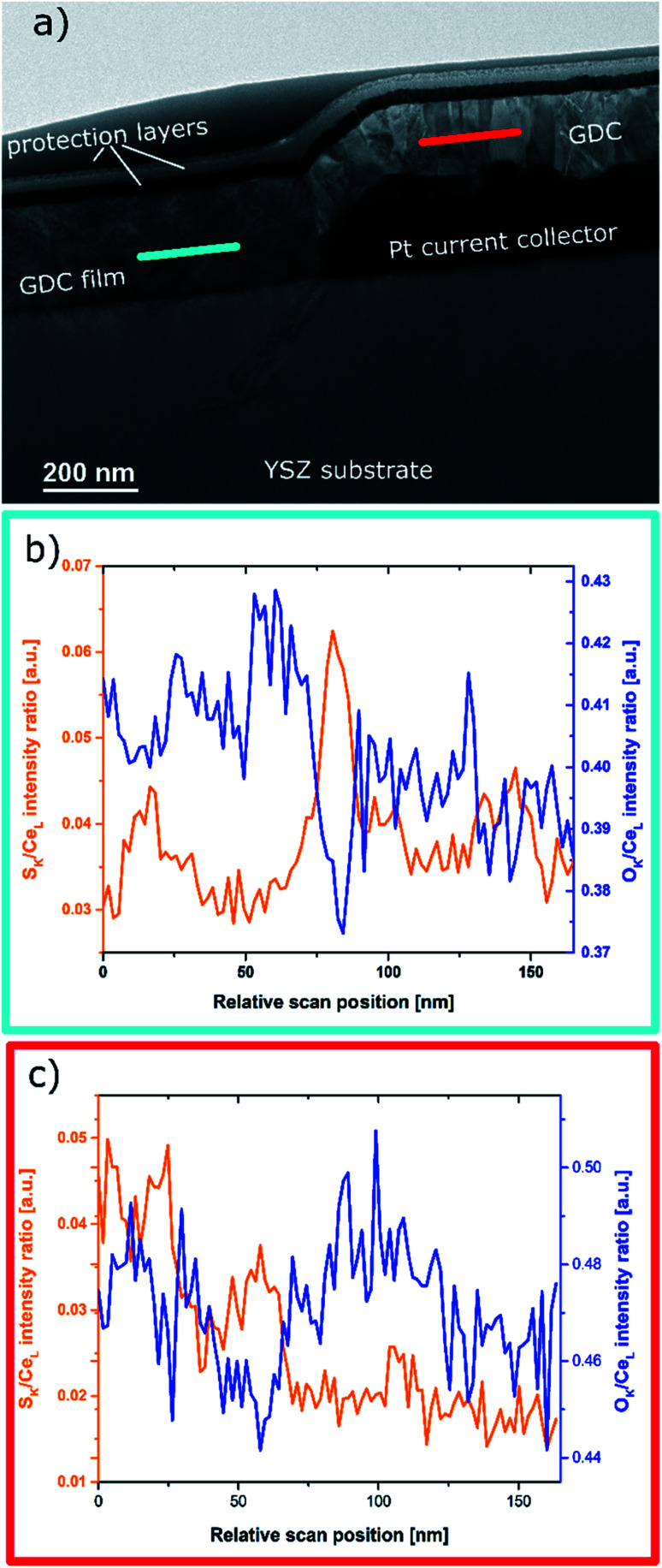
TEM results: (a) brightfield overview of a section presenting the GDC film in direct contact with the YSZ substrate and part of the Pt current collector with GDC on top of it. (b and c) Normalized intensity plots of EDX line-scans in plane of the GDC thin film revealing lateral inhomogeneities of S distribution.

A much better lateral resolution as provided by online-LASIL-ICP-MS can be obtained in transmission electron microscopy (TEM) of very thin sections of the sample. An overview image is given in Fig. 7a, where the cross section prepared by focused ion beam (FIB) milling of the GDC thin film on the YSZ substrate and a part of the Pt current collector is shown. In case of the GDC thin film grown on the Pt current collector (right hand side of Fig. 7a) a polycrystalline columnar structure is clearly visible. The part of the film deposited directly onto the YSZ substrate appears more uniform, but also here 2-dimensional defects such as grain or at least domain boundaries exist, which is also in accordance with previous work.^8^ The positions of two TEM-EDX line scans are marked with two coloured lines in Fig. 7a. The normalized intensity plots of each line scan are depicted in Fig. 7b and c showing lateral inhomogeneities in the S and O distributions. In regions of higher S intensity, the O signal decreases as it can be seen in Fig. 7b at 80 nm relative scan position and in Fig. 7c at *ca.* 25 and 60 nm relative scan position, hence pointing towards grain or domain boundaries being relevant for the formation of the observed pattern. This behaviour further supports our conclusion of an uptake of S and exchange with O especially at the grain boundaries of GDC. For further detailed conclusions, a more extensive quantitative analysis TEM study would be required. Though being indeed of great interest, such a TEM-based elemental analysis is beyond the scope of the present publication.

## 4. Discussion

In the following, we would like to relate the observation that the incorporated sulfur has a strongly detrimental effect on the grain boundary conductivity to the amount of sulfur quantified by online-LASIL-ICP-MS measurements. From the impedance results in this work as well as previous experiments (including TEM)^8^ we can conclude that sulfur accumulates at the grain boundaries of GDC. It is worth noting that the grain boundary is usually divided into a grain boundary core – the atomically sharp plane at which the grains meet – and an oxygen vacancy depleted space charge region, which is the main reason for the electrical resistance in sulfur-free GDC.^38,39^

**Fig. 8:**
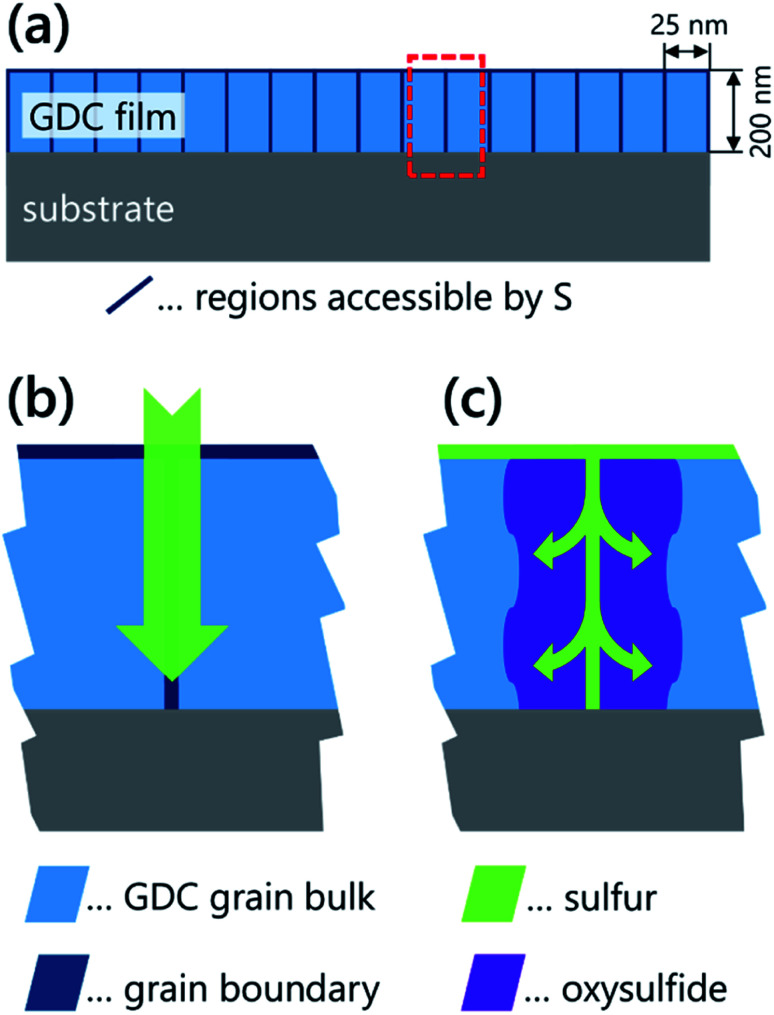
(a) Sketch of GDC thin film cross section. Grain boundaries and surface, which can be accessed by sulfur are highlighted with red colour. (b) Close-up of the sulfur poisoning stages of GDC grain boundaries. Initial situation with sulfur penetrating the grain boundaries and S^2−^ sitting in the grain boundary core. (c) Advanced poisoning with ceriumoxysulfide formation.

For a discussion of the mechanism of sulfur uptake, the amount of incorporated sulfur must be related to the grain boundary area. The GDC film grows columnar, and we can approximate the columns to be square-based with a side length of 25 nm (see Section 2.2 and ref. 21). Such a structure has a specific grain boundary area of 8 × 10^5^ cm^2^ cm^−3^. The total amount of sulphur for the unpolarised film (1.0 ± 0.4 at%) corresponds to about 8 × 10^20^ S atoms per cm^3^, or 10^15^ S atoms per cm^2^ of grain boundary area. It would be possible that these form a relatively dense layer at the grain boundary core, since the density of oxide ions in a (100) GDC plane is about 1.4 × 10^15^. Sulfur most likely also accumulates on the surface (see Fig. 8a), but the surface area of a 200 nm thick GDC film is just 1/16 of its grain boundary area, and therefore cannot be the primary location of the sulfur atoms.

Alternatively, the incorporated sulfide ions might form an oxysulfide phase (*e.g.* Ce_2_O_2_S)^14^ extending from the grain boundary core. For the lowest measured S amount, the width of such an oxysulfide phase would be of the order of the GDC lattice constant (0.542 nm). In case of the highest S amount incorporated upon applying −250 mV (*i.e.* 4.7 ± 0.5 at%), the oxysulfide phase would be almost 2.5 nm thick, which corresponds to about 20 vol% of the entire film. This result also makes it quite clear that such a large amount of sulfur can no longer sit exclusively in the core of the grain boundary thus strongly supporting the assumption of an oxysulfide formation.

From a structural point of view, the formation of such an oxysulfide (Ce_2_O_2_S) phase can be expected to proceed rather easy, because the structure is closely related to that of bixbyite-type Ce(iii)oxide Ce_2_O_3_. The main difference in the oxysulfide phase is that 1/3 of the O^2−^ ions are replaced by S^2−^. Thus, apart from slight lattice distortions, no extensive cation rearrangement is required, and computations predict a low migration barrier for S^2−^ in ceria.^30^ The enhanced reducibility of ceria at surfaces^38,40^ and grain boundaries^21^ is also a probable factor that promotes the formation of the oxysulfide phase starting from the grain boundaries.

Considering these results, we suggest the following tentative mechanism of sulfur incorporation into polycrystalline GDC films: for low H_2_S partial pressures and only moderately reducing atmospheres, sulfur first penetrates the grain boundary core and only minute amounts of S diffuse into the GDC grains without causing a phase change. Such a situation is sketched in Fig. 8b and is commonly called Harrison type C diffusion, in which mass transport takes place almost exclusively *via* the grain boundaries.^41^ This suggestion agrees with the observation in a previous study, where the depth distribution of sulfur in annealed GDC polycrystals was studied by secondary ion mass spectrometry^8^ and the preliminary TEM-EDX measurements included in this work.

If the film is additionally reduced electrochemically, a bulk-like Ce_2_O_2_S phase is then formed, starting from the grain boundaries – see Fig. 8c. Alternatively, a Harrison type B incorporation of sulfur might also be thinkable,^41^ with significant diffusion from the fast grain boundaries into the grains. However, the respective diffusion profile into the grains needs to be rather short to explain the electrical effect exclusively on the grain boundaries, which would thus result in relatively high local sulfur concentrations. Hence, the explanation of an oxysulfide formation at the grain boundaries is a more realistic scenario. Further support for the formation of an oxysulfide phase is derived from the observed solubility of the H_2_S annealed films in the acidic carrier solution in the online-LASIL process (see Section 3.3(c)), since sulfides are commonly more easily soluble in acids than oxides. Dissolution of the oxysulfide phase at the grain boundaries would significantly reduce the stability of the grains and contribute to their detachment.

Since the oxide ion conductivity of Ce_2_O_2_S is very likely much lower than in GDC, the formation of this phase explains the observed decrease in grain boundary conductivity. Nonetheless, we cannot unambiguously state, whether this phase actually grows homogeneously from the grain boundary into the GDC grain, or if this happens rather *via* an island-like growth mechanism. While in case of the first explanation, the ionic current needs to pass through the phase with poor ionic conductivity, the latter would cause a current constriction resistance at the grain boundaries. Electrically a discrimination of both situations is not straightforwardly possible, but the geometric constraints required for the occurrence of a current constriction resistance make this explanation seem rather improbable.^42^ Nevertheless, for unambiguously clarifying the detailed kinetics of sulfur incorporation into polycrystalline GDC films additional work needs to be done in the future.

## 5. Conclusion and outlook

In this work the sulfur uptake into GDC thin films from a H_2_S containing H_2_/H_2_O atmosphere was studied in dependence of the applied electrochemical polarisation. The analytical results are related to the effect of sulfur uptake on the ionic grain boundary resistance of the thin films. The measurement of the in-plane ionic conductivity was done by impedance spectroscopy and the separation of the grain boundary contribution from the grain bulk conductivity was performed by CNLS-fitting the data to the appropriate equivalent circuit. For quantification of the total amount of sulfur incorporated into GDC, online-LASIL-ICP-MS was used as a novel solid sampling technique. Together with the results from electrical measurements, the known amount of S allowed to suggest a tentative mechanism of sulfur incorporation into GDC, which occurs *via* grain boundaries.

To address the specific requirements of sulfur poisoned GDC and the associated analyte combination, several optimization steps were implemented into the online-LASIL-ICP-MS routine. Moreover, the special analytical issues related to the quantification of S were elaborated and a measurement mode with a mass shift reaction with O_2_ as reaction gas was implemented. Various additives for the carrier solution were investigated to prevent memory effects of S. Related to that topic, an adapted online ablation cell was developed to handle pH-sensitive samples as it was the case for degraded GDC thin films. The solubility behaviour of the degraded GDC film gave already some hint about the chemical species formed on the sample surface during the annealing process in H_2_S containing atmosphere. With these improvements, the stoichiometry of Gd and Ce as the main components as well as of S as a minor component of degraded GDC thin films could be successfully determined. In particular, it could be demonstrated that the determination of a few ng of S is feasible with the novel online-LASIL sampling strategy coupled to an ICP-QMS instrument. The smallest determined S quantity was close to 2 ng per measurement and the area ablated in one measurement run was 1.05 mm^2^. Thus, it could be shown that the large field of novel materials containing S as major or minor component is analytically well accessible with this technique and that this approach is well suited for the determination of traces and ultra-traces in thin films. With the adapted cell design, the number of materials potentially being analysed has been significantly enlarged because more unstable materials can be handled as well. Possible memory effects can be prevented in large parts, because additives (*e.g.*, acids or complexation agents) can be added very closely to the actual spot of ablation.

## Author contributions

C. Herzig: investigation (lead), writing – original draft. J. Frank: resources. A. Nenning: investigation (supporting). M. Gerstl: investigation (supporting). A. Bumberger: investigation (supporting). J. Fleig: supervision. A. K. Opitz: conceptualization (equal), data curation, writing – review & editing. A. Limbeck: conceptualization (equal), funding acquisition, writing – review & editing.

## Conflicts of interest

There are no conflicts to declare.

## Supplementary Material

TA-010-D1TA06873C-s001
