# Supplementary material for: Combining electrochemical and quantitative elemental analysis to investigate the sulfur poisoning process of ceria thin film fuel electrodes
Source: J Mater Chem A Mater. 2021 Dec 22;10(4):1840–51. doi: 10.1039/d1ta06873c (PMC8788136; doi:10.1039/d1ta06873c)
Supplement: TA-010-D1TA06873C-s001 [file TA-010-D1TA06873C-s001.pdf]

Electronic Supplementary Material (ESI) for Journal of Materials Chemistry A.
This journal is © The Royal Society of Chemistry 2021

Supplementary Information to

Combining electrochemical and quantitative elemental analysis to investigate the sulfur poisoning process of ceria thin film fuel electrodes

C. Herzig[1], J. Frank[2], A. Nenning[1], M. Gerstl[1], A. Bumberger[1], J. Fleig[1], A.K. Opitz[1*], A. Limbeck[1*]

1- TU Wien, Institute of Chemical Technologies and Analytics, Vienna, Austria

2- TU Wien, Joint Workshop, Technical Chemistry, Vienna, Austria

**Corresponding author: TU Wien, Institute of Chemical Technologies and Analytics, Getreidemarkt 9/164 I²AC, 1060 Vienna, Austria.*

E-mail address: alexander.opitz@tuwien.ac.at, andreas.limbeck@tuwien.ac.at

Ad 2.6. Online-LASIL sampling and quantification procedure

Signal quantification was achieved with standard solutions varying in analyte concentration prepared by mixing 20 vol% nitric acid with single element standards of S, Ce (Specpure®, Alfa Aesar, ThermoFisher, Germany), and Gd (Certipure®, Merck, Germany). The concentration ranged from 14 to 60 ng g^{-1} for S, 0.25 to 2 µg g^{-1} for Gd, and 0.75 to 6 µg g^{-1} for Ce. Since the goal was to determine the S uptake in the form of absolute values instead of molar ratios, it is necessary to determine the amount of liquid standard used for calibration as well. Therefore, a six-way valve with a sample loop was installed before the ablation cell and the sample volume of the loop was gravimetrically determined to be 0.147 +/- 0.002 g (n=6). A flow-chart is depicted in Figure S1. In the load position, the sample loop was filled manually with a syringe and carrier solution 1 is pumped through the six-way valve without further changes. If the position of the valve was set to inject position, the flow of carrier solution 1 transported the standard solution in the sample loop through the whole online-LASIL system to the sample introduction system of the ICP-MS device. The obtained transient signals for standard solutions as well as sample measurements were manually integrated in OriginPro 2020-software (version 9.7.0.185, OriginLab Cooperation) and evaluated subsequently.

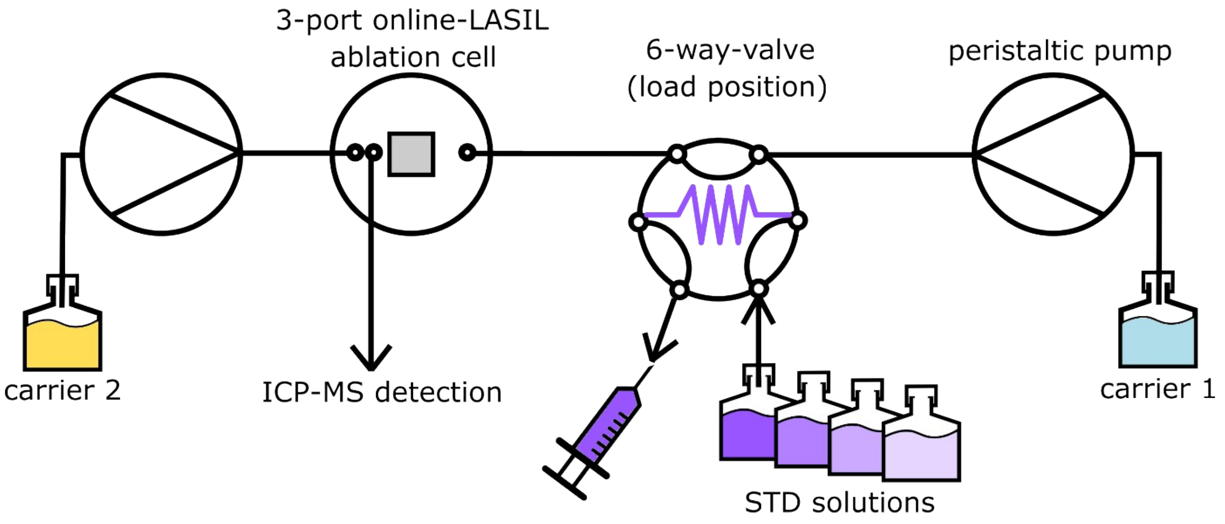

Figure S1: Flow-chart of online-LASIL set-up including a six-way-valve with sample loop for absolute quantification and a three-port online-LASIL ablation cell for pH sensitive samples.

Ad 3.3. Optimization of ICP-MS measurement parameters

Measurement of S using mass shift reaction

In a first optimization step, the appropriate collision cell mode needs to be determined. Therefore, liquid standard solutions containing S (5 to 100 ng g^{-1}) were prepared and measured in both modes with standard settings. To decide which mode works best, the limit of quantification was determined according to DIN 32645. With KED mode, the obtained LOQ was about 20 times higher compared to the LOQ obtained with CCT mode for $^{32}S^{16}O$ (LOQ = 10 +/- 5 ng g^{-1}). Thus, all further optimization steps were carried out using the mass shift of the reaction mode.

For a further improvement of the signal intensity, the reaction gas flow rate and the sample flow rate were investigated, because both parameters influence the sensitivity. If the reaction gas flow rate is too high, the signal intensity might be reduced, because the analyte ions (SO^+) collide with the excess reaction gas and the reaction gas acts like the collision gas in the KED mode. This behaviour is of course unwanted but is linked to the flow rate used for sample introduction, which determines how many S^+-ions are present in the collision cell. In Figure 5, data obtained by varying liquid sample flow rate of a standard solution (25 ng g^{-1} S concentration) and the reaction gas flow rate is depicted. For low sample flow rates, the optimum reaction gas rates are very low. Since the flow rate in our online-LASIL set-up is rather high to minimize particle dispersion, all subsequent experiments were conducted with 1.3 mL

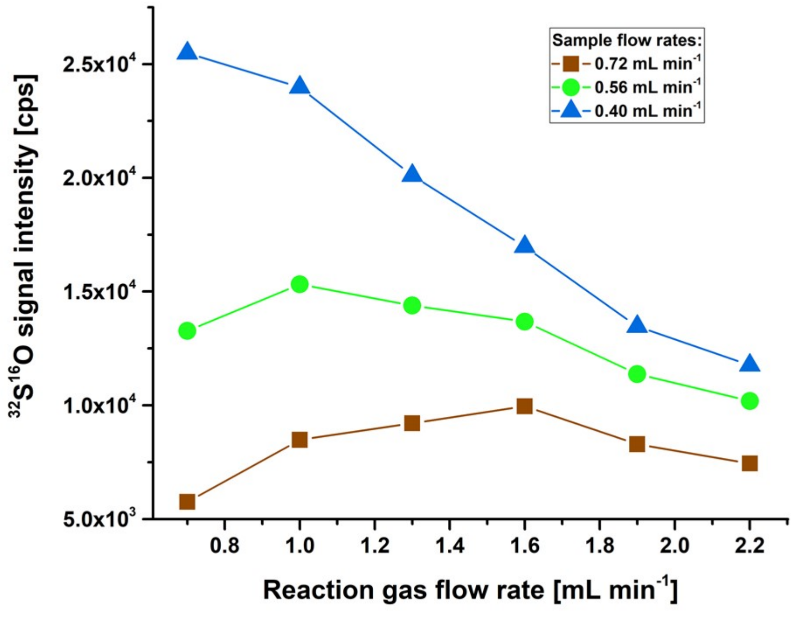

Figure S2: Influence of reaction gas and sample flow rate on $^{32}S^{16}O$ signal intensity.

min^{-1} reaction gas flow rate.

Ad. 3.4 Quantitative analysis of H_2S treated GDC thin films

Formula for calculation of the total GDC mass

Because O is not accessible by ICP-MS, the total mass of the ablated thin film was not directly accessible from the ICP data. However, since for the given experimental conditions, the oxygen content of GDC can be obtained in very good approximation from the cation composition considering oxygen vacancies being the by far dominant compensating point defect [1-3], the total mass of the ablated thin film could be straightforwardly calculated using the following equation:

$$m_O[\mu g] = \frac{(n_{Ce} + n_{Gd}) * 1.95 * M_O}{1000}$$

(1)

Where m_O is the mass of O in microgram, n_{Ce} and n_{Gd} are the ablated amount of Ce and Gd in nmol, 1.95 is the nominal stoichiometry value for O and M_O is the molar mass of O (15.999 g mol^{-1}). The mass fraction of S in GDC is determined by:

$$x_S\,[wt\%] = \frac{m_S}{m_{Ce} + m_{Gd} + m_O} * 100\,\% \qquad (2)$$

Where m_{Ce}, m_{Gd}, m_O, and m_S are the masses of Ce, Gd, O. and S in microgram and x_S is the mass fraction of S in GDC in wt%.

Ad 3.5. Additional analytical analysis

Quantitative XRF measurements

To verify the determined S content in the GDC thin film with a complementary technique, wavelength dispersive X-ray fluorescence (XRF) measurements were conducted on an AXIOS advanced PW 4400/40 spectrometer (Malvern Panalytica, UK), equipped with a 3600 W Rh cathode and multiple gratings for fluorescence detection in the 300-22 000 keV energy range. It is worth mentioning at this point that the information depth of the excited X-rays is much larger than the film thickness, so the entire GDC film is probed and the majority of the fluorescence signal originates from the YSZ substrate. For quantification of the S content, the cross-section corrected sulphur K-line intensity was compared to the Ce L-line intensity, and the quantification routine of the machine's software gave 0.090 wt% S and 4.066 wt% Ce as a result. From this ratio, a sulfur content of 2.9 at% could be calculated for the entire GDC thin film. Since the used XRF instrument does not provide any lateral resolution in the size range necessary for this sample, the obtained result is an average value over the whole sample area.

To be able to compare this value with the ICP-based analysis result, an area-weighted average of the online-LASIL-ICP-MS results was calculated. Therefore, the area of the biased part and the OC part (the latter includes also the gap without Pt current collector) was determined by an image editing software (inkscape 0.91) from an optical microscope image (see Figure S3). The biased part of the sample is 43 % of the whole sample area. With the following equation, the average S content obtained with online-LASIL is calculated for the entire GDC:

$$X_{S,\,LASIL,\,avg}\,[at\%] = X_{S,\,LASIL,\,biased} * 0.43 + X_{S,\,LASIL,\,OC} * 0.57 \qquad (3)$$

Therein, $X_{S,\,LASIL,\,biased}$ and $X_{S,\,LASIL,\,OC}$ are 4.7 at% and 1.0 at% as reported in Figure 6 in the main text, respectively. Hence, an average online-LASIL-ICP-MS measured S content of 2.6 at% in GDC is obtained, which is in excellent agreement with the XRF result.

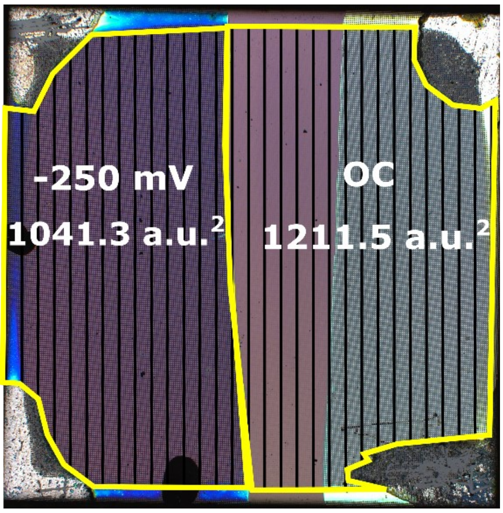

Figure S3: Area determination of the biased and unbiased part of the sample.

Qualitative TEM-EDX measurements

Preparation of the TEM lamella was done by FIB cutting on a Thermo Fisher Scios 2 DualBeam FIB/SEM, operating with a Ga-ion beam at 30 kV accelerating voltage, following a typical in-situ lift-out FIB TEM sample preparation routine [4]. Final low-voltage cleaning of the lamella was performed at 5 kV and 2 kV. Transmission electron microscopy was performed on a FEI TECNAI F20 equipped with an EDAX APOLLO XII EDX detector. For qualitative elemental analysis revealing lateral inhomogeneities in the S distribution, line-scans in the GDC film were recorded. For this means, the intensities of the O_K, S_K, Ce_L, and Gd_L emission lines were measured as a function of the position. The resulting lateral profiles are shown in Section 3.5 of the main text.

References:

1. Otake, T., et al., *Nonstoichiometry of Ce1−XYXO2−0.5X−δ (X=0.1, 0.2).* Solid State Ionics, 2003. **161**(1): p. 181-186.
2. Wang, S., et al., *Nonstoichiometry of Ce0.9Gd0.1O1.95−x.* Solid State Ionics, 1998. **107**(1): p. 73-79.
3. Lai, W. and S.M. Haile, *Impedance Spectroscopy as a Tool for Chemical and Electrochemical Analysis of Mixed Conductors: A Case Study of Ceria.* Journal of the American Ceramic Society, 2005. **88**(11): p. 2979-2997.
4. Langford, R.M. and M. Rogers, *In situ lift-out: Steps to improve yield and a comparison with other FIB TEM sample preparation techniques.* Micron, 2008. **39**(8): p. 1325-1330.
